# The Value of Artificial Intelligence-Assisted Imaging in Identifying Diagnostic Markers of Sarcopenia in Patients with Cancer

**DOI:** 10.1155/2022/1819841

**Published:** 2022-03-29

**Authors:** Ying-Tzu Huang, Yi-Shan Tsai, Peng-Chan Lin, Yu-Min Yeh, Ya-Ting Hsu, Pei-Ying Wu, Meng-Ru Shen

**Affiliations:** ^1^Division of Medical Oncology, Department of Oncology, National Cheng Kung University Hospital, College of Medicine, National Cheng Kung University, Tainan, Taiwan; ^2^Department of Medical Imaging, National Cheng Kung University Hospital, College of Medicine, National Cheng Kung University, Tainan, Taiwan; ^3^Division of Hematology, Department of Internal Medicine, National Cheng Kung University Hospital, College of Medicine, National Cheng Kung University, Tainan, Taiwan; ^4^Department of Obstetrics and Gynecology, National Cheng Kung University Hospital, College of Medicine, National Cheng Kung University, Tainan, Taiwan

## Abstract

Sarcopenia is defined as the loss of skeletal muscle mass and muscle function. It is common in patients with malignancies and often associated with adverse clinical outcomes. The presence of sarcopenia in patients with cancer is determined by body composition, and recently, radiologic technology for the accurate estimation of body composition is under development. Artificial intelligence- (AI-) assisted image measurement facilitates the detection of sarcopenia in clinical practice. Sarcopenia is a prognostic factor for patients with cancer, and confirming its presence helps to recognize those patients at the greatest risk, which provides a guide for designing individualized cancer treatments. In this review, we examine the recent literature (2017-2021) on AI-assisted image assessment of body composition and sarcopenia, seeking to synthesize current information on the mechanism and the importance of sarcopenia, its diagnostic image markers, and the interventions for sarcopenia in the medical care of patients with cancer. We concluded that AI-assisted image analysis is a reliable automatic technique for segmentation of abdominal adipose tissue. It has the potential to improve diagnosis of sarcopenia and facilitates identification of oncology patients at the greatest risk, supporting individualized prevention planning and treatment evaluation. The capability of AI approaches in analyzing series of big data and extracting features beyond manual skills would no doubt progressively provide impactful information and greatly refine the standard for assessing sarcopenia risk in patients with cancer.

## 1. Introduction

Sarcopenia was first introduced by Dr. Irwin Rosenberg in 1989, who described it as “age-related loss of skeletal muscle” [1]. It was initially regarded as the progressive decline in skeletal muscle mass, muscle strength, and physical performance associated with aging [2], but the definition and management of sarcopenia have expanded in recent years. In today's broader view, besides associations with aging, the shared risk factors for development of sarcopenia include chronic diseases, nutrition deficiencies, physical inactivity, hormonal changes, insulin resistance, loss of the neurons that stimulate muscle, and fat infiltration into muscle [3]. Among possible comorbidities, malignancy is a major category of disease-related sarcopenia. The causes of muscle loss in patients with cancer are multifactorial, especially in older adults [4]. Gender differences have been found in the prevalence of sarcopenia for people younger than 70 years and those older than 80 years; sarcopenia is diagnosed more often in women in those aged <70 years, while among those aged >80 years, more men will have sarcopenia than women [5]. This gender difference is clearly influenced by age, and sarcopenia must be considered when evaluating people of all ages who have cancer.

The etiology of sarcopenia in patients with cancer may vary between different ages and genders and can be associated with genetic predisposition, underlying comorbidities, reduced physical performance, and age-related declines in various hormones. Cancer-induced inflammatory cytokines and anorexia that cause decreased protein intake and synthesis and increased protein degradation may also be markers of sarcopenia in cancer patients. Treatment-related causes may include the side effects of chemotherapy, surgery, or radiotherapy [4, 6].

Sarcopenia is a prognostic factor for patients with cancer, and confirming its presence helps to recognize those patients at the greatest risk and to guide individualized cancer treatment [7]. The diagnosis of sarcopenia is determined through the assessment of body composition (analysis of adipose and muscle tissue components), and recently, artificial intelligence- (AI-) assisted image measurement is being used to facilitate the detection of sarcopenia in clinical practice [8].

The purpose of this review was to synthesize current information in recent studies addressing AI-assisted imaging assessment of body composition and sarcopenia, particularly to gain a clearer understanding of the mechanism and the importance of sarcopenia in cancer and its diagnostic image markers and interventions for sarcopenia in the medical care of patients with cancer.

## 2. Literature Review

We searched the recent literature in PubMed from 2017 to 2021 using (“deep learning”[MeSH Terms] OR (“deep”[All Fields] AND “learning”[All Fields]) OR “deep learning”[All Fields]) AND (“sarcopenia”[MeSH Terms] OR “sarcopenia”[All Fields]). A total of 28 articles addressing AI-assisted imaging assessment of body composition and sarcopenia were found, of which 20 reporting DICE coefficients were finally included for review. They are discussed below along with other supportive studies for background, focusing on cancer-related sarcopenia and the current status of AI-assisted imaging in the evaluation of sarcopenia in cancer patients.

### 2.1. The Definition/Mechanism of Sarcopenia in Cancer Patients

Complex metabolic pathways are involved in the development process of sarcopenia. Several discriminating metabolites have been identified and investigated as potential biomarkers for the presence of sarcopenia. For example, one study demonstrated that low levels of plasma lysophosphatidylcholine 18 : 2 predict a greater decline of gait speed in older adults [9]. Another study reported that increased asparagine, aspartic acid, citrulline, ethanolamine, glutamic acid, sarcosine, and taurine are found in older adult patients with sarcopenia [10]. As for patients with cancer, a serum and urine metabolomics study found that cancer-related metabolic reprograming may represent a distinct diagnostic model [11].

### 2.2. Diagnostic Image Markers for Sarcopenia

In clinical practice, assessment techniques for sarcopenia include handgrip strength to measure muscle strength and gait speed and chair stand tests to evaluate physical performance [12]. Bioimpedance analysis and dual-energy X-ray absorptiometry are the most common diagnostic tools for confirmation of muscle quantity and quality [13]. In the field of oncology, the use of abdominal computed tomography (CT) to measure body composition helps to identify sarcopenia in patients with cancer by providing precise and simplified data for describing sarcopenia and its correlation with clinical factors [14]. Thus, the performance of routine abdominal CT at cancer diagnosis, posttreatment evaluation, and regular follow-up provides the means for gauging body composition throughout the course of cancer.

**Figure 1:**
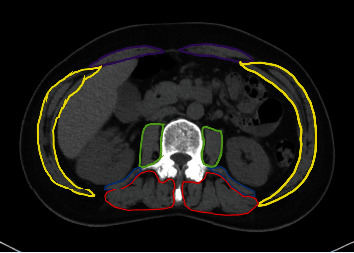
The muscle groups for the skeletal muscle index consist of psoas major (green), quadratus lumborum (blue), erector spinae (red), and abdominal wall muscles (transversus abdominis muscle, internal and external oblique muscle (yellow), and rectus abdominis (purple)).

The cross-sectional area (CSA) of muscle tissue at the level of the 3^rd^ lumbar spine (L3) provides reproducible evaluation of muscle size in cancer patients without the need for additional examinations. The measurements collected from a single slice CT image reveal solid evidence that correlates strongly with whole-body adipose tissue and skeletal muscle [15–17]. The common method is to manually draw the total CSA of all muscle groups at L3 or to quantify the CSA using thresholds of Hounsfield units (HU) from -29 to 150 for skeletal muscle using the available software [18]. The third lumbar vertebra, L3, is chosen because it is the current gold standard for quantification of muscle mass by obtaining parameters from the analysis of a single-slice CT scan [19]. The cross-sectional skeletal muscle area (SMA) calculated at the level of L3 can correctly estimate total body muscle mass [17]. A review has shown that attempts to use alternate vertebral levels to L3 (cervical, thoracic, and lumbar CT slices) for evaluating SMA in cancer patients have shown no validation of whole-body skeletal muscle mass in various types of cancer (lung, head, and neck) and a lack of consensus [20]. The skeletal muscle index (SMI, cm^2^/m^2^) is calculated by dividing the CSA by the square of body height with various cut-off values according to gender and different body mass index (BMI≧25.0 or <25.0) [21]. The formula used was SMI = L3 skeletal muscle CSA (cm^2^)/height^2^ (m^2^). The muscle groups for SMI consist of psoas major, paraspinal muscle, and abdominal wall muscles (Figure 1). The solitary muscle indices such as psoas muscle index (PMI) and paravertebral muscle index (PSMI) also achieve good performance for sarcopenia evaluation [16, 22, 23]. The CT-derived measurement of muscle mass is usually evaluated using the method with thresholds of HU from -29 to 150 that will limit the evaluation of myosteatosis (fat infiltrates into muscle) technologically. The patients with higher BMI had greater SMI but lower skeletal muscle density (SMD) [24, 25]. In the future, CT-derived measurement of muscle mass (area) and quality (myosteatosis) could be achieved with fully automated segmentation for contouring of muscle groups using deep learning systems [26].

### 2.3. The Importance of Sarcopenia in Patients with Cancer

The presence of sarcopenia in older adults may manifest as impaired daily function, disability, increased falls, risk of fractures, loss of independence, poorer quality of life, increased mortality, and high healthcare expenditures [27–31]. In patients with malignancies, sarcopenia is strongly associated with poor oncologic outcomes. A meta-analysis of 4262 participants with ovarian cancer revealed a significant association between the SMI and overall survival (OS) (*P* = 0.007; hazard ratio (HR): 1.11; 95% confidence interval (CI): 1.03-1.20) [32]. Another meta-analysis of 5497 participants with breast cancer reported similar result (pooled HR: 1.71; 95% CI: 1.25-2.33) [33].

Sarcopenia is also an independent predictor of treatment-related toxicities, including surgical complications, prolonged hospitalization, and more adverse effects of chemotherapy. A cohort study of 234 patients undergoing liver resection for malignant tumors demonstrated that sarcopenic patients had longer hospital stays (10 days vs. 6-8 days; *P* < 0.001) and more readmission (8.8% vs. 0-7.7%; *P* = 0.02) than those without sarcopenia [34]. A study of 533 patients with nonmetastatic colon cancer receiving a FOLFOX regimen reported that lower muscle mass is associated with early discontinuation of chemotherapy (odds ratio (OR): 2.34; 95% CI: 1.04-5.24; *P* = 0.03), treatment delay (OR: 2.24; 95% CI: 1.37-3.66; *P* = 0.002), and dose reduction (OR: 2.28; 95% CI: 1.19-4.36; *P* = 0.01) [35].

Body weight or BMI as an indication of body composition was previously used to predict the clinical outcomes of patients with cancer [36, 37]. Emerging evidence suggests that SMI correlates better with negative outcomes and complications than does BMI. A study of 484 patients with pancreatic cancer showed that the changes in BMI during chemotherapy did not have an impact on OS in patients with maintained SMI values (*P* = 0.750), while decreases in SMI were associated with poor OS in patients with maintained BMI (HR: 1.502; *P* = 0.002) [38]. This can be explained by the fact that patients with the same BMI may have different SMI values due to different amounts of muscle mass and differences in the level of fat infiltration. Similarly, patients with the same body surface area (BSA) but different SMI value receiving the same dose of chemotherapy may have different severity of adverse effects [39, 40].

### 2.4. Interventions for Sarcopenia within the Medical Care of Patients with Cancer

The prevalence of sarcopenia in patients with cancer ranges widely from 16% to 71%, depending on the definition in various study settings [7]. The understanding of the presence and the progression of sarcopenia helps to identify high-risk patients and guide the development of treatment plans. Since sarcopenia is significantly associated with treatment-related toxicity [34, 35], the dose titration of chemotherapy, the intensity of surgical intervention, and the schedule of postoperative care should be carefully assessed in sarcopenic patients. For the impact of sarcopenia on oncologic outcomes, it also implies the physician about the disease explanation, prognosis expectation, and treatment decision-making.

The interventions for sarcopenia in patients with cancer include nutritional support, resistance exercise, and specific treatments for sarcopenia and the underlying disease [6, 41–45]. Many studies support the use of nutritional supplements [45], pharmacologic agents to increase muscle mass [44], and exercise programs [42]. Some studies show conflicting results for interventions for increasing muscle mass [6, 41], and the impact of those interventions on clinical outcomes is still being investigated. Prospective studies on interventions for sarcopenia in patients with cancer are limited.

### 2.5. Medical AI Perspectives in the Diagnosis of Sarcopenia

The present review identified a total of 20 articles reporting DICE similarity coefficient scores [16, 19, 46–63].  Table 1 lists the included articles with the population characteristics and segmentation approaches. The reported CT threshold and DICE coefficients of these included studies ranged between 0.93 and 0.98 (Table 2), indicating great promise in the clinical application of AI-assisted imaging. However, as shown in Tables 1 and 2, there is currently no standardized methodology for assessment of sarcopenia. The slicing regions, methods of segmentation, tissues of interest, and ground truth applied varied between the studies. A total of 18 articles used deep learning methods to perform automated segmentation (16 applied fully convolutional networks (FCN) or U-Net, and 2 used ResNet-18). The region of segmentation varied across different systems, but the L3-level axial slice was analyzed most frequently due to its strong correlation with whole-body composition [19]. As reference for segmentation (ground truth), 10 studies reported use of a combination of automated or semiautomated commercial segmentation software or cloud-based annotation tool with manual correction; 1 study specified that expert-labeled annotation was used as ground truth; details of the ground truth reference was not specified in the remaining articles (Table 1). Thirteen studies reported CT threshold HU values. However, the CT threshold is likely affected by whether or not contrast medium was used for imaging. Of the 20 articles reporting DICE scores, 10 articles reported DICE coefficients for skeletal muscle only; in the other 10 articles, tissues including visceral adipose tissue, subcutaneous adipose tissue, and intermuscular adipose tissue were also analyzed. Most of the articles reported training and testing cohort results only; 7 studies performed independent validations (internal or external) (Table 2).

In the evaluation of sarcopenia, abdominal musculature segmentation is accomplished using deep learning with a DICE similarity coefficient of 0.93-0.98 [46, 48]. Successful individual segmentation of different muscle groups for SMI are achieved using a DICE similarity coefficient of 0.82-0.95, consisting of psoas major, quadratus lumborum, erector spinae (paraspinal muscle), and abdominal wall muscles (transversus abdominis muscle, internal and external oblique muscle, and rectus abdominis) [47]. The highly accurate segmentation of individual muscle groups provides an opportunity to assess muscle mass and myosteatosis separately. The area of muscular CSA could be reserved for mass evaluation. Using the cut point of CT HU inside the segmented CSA is aimed at assessing myosteatosis [64]. The CT-derived measurement of myosteatosis is associated with cut points of muscle attenuation less than 41 or less than 33 HU, which is consistent with the most common threshold for low-density muscle (0-30 HU) [64]. Knowledge about changes in body composition during cancer treatments and the disease course is currently lacking. The lack of standardized assessment method to determine muscle mass in cancer patients is evident from the varied cut-off values used in different studies, even for the same cancer type (as reviewed by Rier et al. [65] in 2016). The variations in cut-off value between the same cancer types likely have resulted from the different population characteristics between studies including age, BMI, disease severity, and different methods of evaluation [65]. Recent studies have focused on developing reference diagnostic cut-off values among the normal population. For people under 60 years old, the cut-off SMI value ranged between 40 and 45 in male and 30 and 35 in female (Supplement Table 1) [66–72]. However, the population characteristics were different between these studies, and determination of normal reference cut-off values for different population characteristics using larger series of data via an AI-assisted approach may fasten the development of standardized assessment. AI-assisted body composition measurement would increase the accuracy and efficiency of the sarcopenia evaluation and provides a trend of standardization by which the serial changes in cancer-related sarcopenia are explored [26].

## 3. Conclusion

In conclusion, the presence of sarcopenia is represented by prognostic and predictive values in patients with cancer. AI-assisted image analysis is a reliable automatic technique for segmentation of abdominal adipose tissue with the potential to improve diagnosis of sarcopenia and facilitates identification of oncology patients at the greatest risk, supporting individualized prevention planning and treatment evaluation. The capability of AI approaches in analyzing series of big data and extracting high-level abstractions beyond manual skills would no doubt progressively provide impactful information and greatly refine the standard for assessing sarcopenia risk in patients with cancer.

## Figures and Tables

**Table 1:** Summary of segmentation methods.

	Author (year)	Population	Mean age (year)	Localization	Neural network	Segmentation algorithm	Segmentation ground truth
1	Ackermans (2021) [19]	Cancer surgery cases, colorectal, ovarian, pancreatic cancers (training); polytrauma patients (testing)	Testing: 74	L3 muscle (L3M), intramuscular adipose tissue (IMAT), visceral adipose tissue (VAT), and subcutaneous adipose tissue (SAT)	DLNN	2D U-Net	Manual segmentation using software (TomoVision software “sliceOmatic”)
2	Borrelli (2021) [51]	Lymphoma (training) Prostate cancer (testing)	Training: 61 Testing: 67	L3	CNN	RECOMIA platform U-Net	Manual segmentation using cloud-based annotation tool (RECOMIA, http://www.recomia.org)
3	Castiglione (2021) [52]	Pediatric patients	0-18	Skeletal muscle area at the L3 level; 12-section or 18-section MIP images	CNN	U-Net	Manual segmentation
4	Amarasinghe (2021) [49]	Non-small-cell lung cancer	67	Skeletal muscle at the L3 vertebra	CNN+DL	2.5D U-Nets	Manual segmentation based on the Alberta protocol
5	Kim (2021) [58]	Gastric cancers receiving gastrectomy	60.4	L3	CNN	ResNet-18	Manual segmentation with software (Aquarius 3D workstation, TeraRecon)
6	Magudia (2021) [61]	Pancreatic adenocarcinoma	52	L3	CNN	DenseNet architecture model to predict spatial offset U-Net architecture model for segment	Manual segmentation with software internal data set: sliceOmatic (TomoVision, Magog, Canada); external data set: OsiriX (Pixmeo, Bernex, Switzerland)
7	Koitka (2021) [59]	Individuals with abdominal CT scans (unknown patients)	Training: 62.6 Test: 65.6	Whole abdomen and not just on L3 slices	CNN	Multiresolution U-Net 3D	For annotation, the ITK Snap software (version 3.8.0) was used. Region segmentation was performed manually with a polygon tool
8	Hsu (2021) [57]	Pancreatic cancer	67	L3	CNN	ResNet-18 model for slice 2D U-Net to segment	Manual annotated, expert labeled
9	Zopfs (2020) [16]	The Cancer Imaging Archive's collection “CT Lymph Nodes” and the institutional picture archiving and communication system	62	Containing the abdomen and images above (cranial) and below (caudal) this region	DCNN	U-Net	Manual segmentation
10	Edwards (2020) [54]	Adult patients	18-75	L3	CNN	Supervised U-Net	Manual segmentation
11	Hemke (2020) [56]	200 subjects	49.9	Pelvic content	DCNN	U-Net	Manual segmentation using manual and semiautomated thresholding using the Osirix DICOM viewer (version 6.5.2, http://www.osirix-viewer.com/index.html)
12	Burns (2020) [47]	102 sequential patients	68	L1-L5	CNN	U-Net	Annotation utilizing ITK-SNAP software. Region segmentation was performed manually
13	Paris (2020) [48]	Critically ill, liver cirrhosis, pancreatic cancer, and clear cell renal cell carcinoma patients, renal and liver donors	Training/validation: 52.6 Test: 53.9	L3	DCNN	Adapt U-Net	Manually segmented by using SliceOmatic (TomoVision, Montreal, Canada, version 4.2, 4.3, and 5.0)
14	Blanc-Durand (2020) [46]	Unknown subjects	N/A	L3	DCNN	2D U-Net	Manually annotated using the public freeware 3DSlicer
15	Park (2020) [62]	Gastric cancer, pancreatic cancer, and sepsis and healthy individuals	Training: 56.1 Internal validation: 56.6 External validation: 61.1	L3	CNN	FCN-based	Semiautomated segmentation software (AsanJ-Morphometry) followed by manual correction
16	Barnard (2019) [50]	Older adults, who were current or former smokers	71.6	T12	CNN	U-Net	Manual segmentation using Mimics software (Materialise, Leuven, Belgium)
17	Graffy (2019) [55]	Asymptomatic adults	57.1	L3	CNN	U-Net	Manual segmentation
18	Dabiri (2019) [53]	Data from Cross Cancer Institute (CCI), University of Alberta, Canada	N/A	L3 and T4	CNN	FCN with VGG16	Manual segmentation using Slice-O-Matic V4.3 software (TomoVision, Montreal, Canada)
19	Lee (2017) [60]	Patients with lung cancer	63	L3	CNN	FCN of ImageNet pretrained model	Semiautomated threshold-based segmentation, followed by manual correction
20	Shephard (2015) [63]	N/A	N/A	N/A	N/A	N/A	

L3M: L3 muscle; IMAT: intramuscular adipose tissue; VAT: visceral adipose tissue; SAT: subcutaneous adipose tissue; DLNN: deep learning neural network; CNN: convolutional neural network; MIP: maximum intensity projections; DL: deep learning; DCNN: deep convolutional neural network; N/A: not available; FCN: fully convolutional network.

**Table 2:** Summary of review of 20 articles reporting the CT threshold, DICE similarity coefficient scores, and study limitations.

	Author (year)	Population	Patients (*N*)	CT threshold (HU value)	DICE score	Limitations
1	Ackermans (2021) [19]	Cancer surgery cases, colorectal, ovarian, pancreatic cancers (training); polytrauma patients (testing)	Training: 3,413 Testing: 233	Muscle: -29 to +150 HU	L3M: 0.926 (0.866–0.959)^†^ VAT: 0.951 (0.888-0.974)^†^ SAT: 0.953 (0.916-0.975)^†^	(1) This algorithm systematically overestimates muscle area (2) Overlapping adjacent internal organs with muscle and CT Hounsfield units being similar between some organs and muscle also lead to a degree of misidentification as muscle
2	Borrelli (2021) [51]	Lymphoma (training) Prostate cancer (testing)	Training: 50 Testing: 74	SAT: -190 to -30 HU Muscle: -30 to +150 HU	SAT: mean = 0.96 Muscle: mean = 0.94	(1) Used manual segmentations of SAT and muscle in a single CT slice at the L3 level to validate the AI-based method (2) In 9% of the cases, a manual correction was needed due to difficulty to detect T11 by the AI-based tool (3) The VAT compartment was not included in the analysis
3	Castiglione (2021) [52]	Pediatric patients	Training: 296 Testing:74	N/A	DSC: 0.93 ± 0.03^‡^	(1) The limited availability of ground truth data for a pediatric population (2) Did not attempt to account for variant anatomy, including patients who only had 11 rib-bearing thoracic-type vertebral bodies and patients who had transitional vertebrae at the lumbosacral junction
4	Amarasinghe (2021) [49]	Non-small-cell lung cancer	Training and validation: 66 Testing (internal): 42	Muscle: -29 to +150 HU	5-fold cross-validation: mean = 0.92 Internal test: mean = 0.96	(1) In some cases, with very low SM area, the model tends to misclassify other organs as belonging to skeletal muscle (2) Observed limited benefit of data augmentation apart from flipping and addition of Gaussian noise, which may suggest limited variability in the validation set (3) Systematic difference between the manual and automated segmentation occurred when the patient was scanned with arms down (4) Specific image normalization methods and model parameter tuning are needed to extend our method to other modalities, including diagnostic quality CTs and magnetic resonance imaging (MRI)
5	Kim (2021) [58]	Gastric cancers receiving gastrectomy	840	Skeletal muscle: -29 to +150 HU Adipose tissue: -190 to -30 HU	ICC for SMA: 0.604	(1) Not all of the automatically derived segmentation data could be used
6	Magudia (2021) [61]	Pancreatic adenocarcinoma	Training: 421 Validation: 94 Testing (internal): 89	Muscle: -29 to +150 HU Fat: -190 to -30 HU	Testing (internal): Muscle: 0.97 ± 0.03^‡^ SF: 0.98 ± 0.02^‡^ VF: 0.95 ± 0.10^‡^	(1) Although the aim was to focus on patients without a major cardiovascular or oncologic diagnosis at the time of imaging, the included patients underwent imaging for a reason and may have been less healthy than the average American adult (2) Volumetric BC segmentation, which required large-scale collection of many manually segmented CT slices per patient examination for model training and validation
7	Koitka (2021) [59]	Individuals with abdominal CT scans (unknown patients)	Training: 32 Validation: 8 Testing: 10	Muscle: -29 to +150 HU Fat: -190 to -30 HU	Mean = 0.9553	The collected dataset was from slice thickness of 5 mm
8	Hsu (2021) [57]	Pancreatic cancer	Experiment 1: (i) Training: 28 (ii) Testing: 12 Experiment 2a: (i) Training: 28 (ii) Testing: 12 Experiment 2b: (i) Training: 56 (ii) Testing: 12 Clinical application: 136	-150 to 250 HU	Experiment 1: Training: Muscle: 0.92 [0.91, 0.93]^&^ SF: 0.93 [0.90, 0.95]^&^ VF: 0.89 [0.86, 0.92]^&^ Testing: Muscle: 0.83 [0.80, 0.86]^&^ SF: 0.90 [0.88, 0.93]^&^ VF: 0.76 [0.70, 0.81]^&^ Experiment 2: Muscle: 0.85 [0.83, 0.88]^&^ SF: 0.92 [0.91, 0.93]^&^ VF: 0.80 [0.77, 0.83]^&^	(1) There was a generalization gap across datasets when tested on local pancreatic cancer data (2) Analysis was restricted to a single slice using a 2D U-Net architecture (3) All image labels were performed by two radiologists, and disagreement was solved by consensus, without documenting the disagreement systematically
9	Zopfs (2020) [16]	The Cancer Imaging Archive's collection “CT Lymph Nodes” and the institutional picture archiving and communication system	Training cohort: (i) Training: 72 (ii) Validation: 14 Validation cohort: (A) 24 patients used to assess the consistency of the developed method (B) 39 patients underwent concurrent SDCT and BIA	Muscle: 15 to 200 HU Fat: -200 to -50 HU	Validation: 0.95 Muscle and SF: 0.99 VF: 0.98	(1) The included patients may be subject to a selection bias (2) This study used iodine maps derived from SDCT, a dual-layer based method of dual-energy CT (3) While parenchymatous organs are reliably excluded due to their clearly higher perfusion, portions of the bowel wall, feces, gall bladder, and bile may be misclassified as muscle (4) This study used an independent test set of patients with repetitive examinations to validate the whole chain of DCNN and thresholding in addition to independent test sets for every step in DCNN-based processing
10	Edwards (2020) [54]	Adult patients	Training: 61 (682 images) Validation: 3 (85 image) Testing: 5 (137 images)	N/A	Training: 0.92 ± 0.032^‡^ Validation: 0.92 ± 0.035^‡^ Testing: 0.92 ± 0.024^‡^	(1) The limitation to this approach is undermining significant muscle mass changes that may be characteristic of sarcopenia (2) Better understanding of what determines a “significant” skeletal abdominal muscle mass changes must be understood further to introduce postprocessing image correction in the clinical setting
11	Hemke (2020) [56]	200 subjects	Training: 180 Testing: 20	Muscle -29 to +150 HU SAT: -190 to -30 HU	Miscellaneous intrapelvic content: 0.98 SAT: 0.97 Muscle: 0.95 IMAT: 0.91 Bone: 0.92	(1) The model being trained using a single standardized slice at the pelvis (2) Cohort trending towards overweight BMIs, with possible variations in accuracy for subjects with very low BMI
12	Burns (2020) [47]	102 sequential patients	Training: 51 Testing: 51	N/A	Train: abdominal muscle Third lumbar vertebrae: 0.953 ± 0.015^‡^ Fourth lumbar vertebrae: 0.953 ± 0.011^‡^ Test: abdominal muscle Third lumbar vertebrae: 0.938 ± 0.028^‡^ Fourth lumbar vertebrae: 0.940 ± 0.026^‡^ Train: psoas muscle Third lumbar vertebrae: 0.942 ± 0.040^‡^ Fourth lumbar vertebrae: 0.951 ± 0.037^‡^ Test: psoas muscle Third lumbar vertebrae: 0.939 ± 0.028^‡^ Fourth lumbar vertebrae: 0.946 ± 0.032^‡^	Inclusion criterion of 59 years and older
13	Paris (2020) [48]	Critically ill, liver cirrhosis, pancreatic cancer, and clear cell renal cell carcinoma patients, renal and liver donors	Training and validation: 804 Testing: 89	Muscles: -29 to +150 HU IMAT: -190 to -30 HU VAT: -150 to -50 HU SAT: -190 to -30 HU	Muscle: 0.983 ± 0.013^‡^ IMAT: 0.9 ± 0.034^‡^ VAT: 0.979 ± 0.019^‡^ SAT: 0.986 ± 0.016^‡^	
14	Blanc-Durand (2020) [46]	Unknown subjects	Training: 1,025 Testing: 500	Muscle: -29 to 150 HU	Testing: 0.97 ± 0.02^‡^	(1) Independent cohort would be mandatory to validate the algorithm (2) Because of the anonymization process, height and weight were not available for stratification
15	Park (2020) [62]	Gastric cancer, pancreatic cancer, and sepsis and healthy individuals	Training: 467 (883 images) Validation (internal): 308 (426 images) Validation (external): 171 (171 images)	Muscle: -29 to +150 HU Fat tissue: -190 to -30 HU	Internal validation:0.96 ± 0.03^†^ Muscle: 0.96 SF: 0.97 VF: 0.97 External validation: 0.97 ± 0.01^†^ Muscle: 0.97 SF: 0.97 VF: 0.97	(1) Patient recruitment process was not consecutive; this may have resulted in selection bias (2) External validation was performed using data from a limited number of subjects from only two institutions; large-scale external validation might be necessary
16	Barnard (2019) [50]	Older adults, who were current or former smokers	Training: 1,875 Testing: 209	Muscle: -29 to +150 HU	Testing: 0.94 ± 0.04^#^	(1) The CT slice cannot be automatically selected (2) Only low-dose CT scans were used
17	Graffy (2019) [55]	Asymptomatic adults	8037	N/A	DSC: 0.938 ± 0.028^‡^	(1) All cases were derived from a single medical center on symptomatic adults employing scanners from a single CT vendor, with a fairly uniform unenhanced protocol (2) Did not correlate muscle segmentation values with downstream adverse clinical outcomes
18	Dabiri (2019) [53]	Data from Cross Cancer Institute (CCI), University of Alberta, Canada	Dataset-1: 1075 images Dataset-2: 5101 images Dataset-3: 3003 images	Muscle: -29 to +150 HU	From 0.9713 to 0.9912 (mean ranges)	(1) The performance of the model depends profoundly on the provided ground truth labels and their accuracy. Mistakes in the labeling process will transmit through to the network's definition of skeletal muscle tissue and can result in the model making the same mistakes. Availability of standardized labels using a common protocol would help mitigate the errors due to protocol differences
19	Lee (2017) [60]	Patients with lung cancer	Entire cohort: 400 (250 training images and ground truth)	Skeletal muscle CSA: -29 to +150 HU	DSC: 0.93 ± 0.02^‡^	(1) The network statistically tends to underestimate muscle CSA, probably due to a combination of overlapping HUs between muscle and adjacent organs and variable organ textural appearance. On the other end of the spectrum, segmentation is also confused by the radiographic appearance of edema particularly in obese patients, which has a similar HU range to muscle, leading to higher CSA than expected. Extensive edema tends to occur in critically ill patients, leading to potentially falsely elevated CSA in patients actually at higher risk for all interventions (2) The network should be trained to segment CT examinations performed without intravenous contrast and ultralow radiation dose
20	Shephard (2015) [63]	N/A	N/A	N/A	Normal liver: DSC = 0.93 Enhancing tumor DSC = 0.74 Necrotic tumor: DSC = 0.72	N/A

L3M: L3 muscle; IMAT: intramuscular adipose tissue; VAT: visceral adipose tissue; SAT: subcutaneous adipose tissue; SMA: skeletal muscle area; SF: subcutaneous fat; VF: visceral fat; CSA: cross-sectional area; DSC: DICE similarity coefficient; ICC: intraclass correlation coefficient; SDCT: spectral detector computed tomography; BIA: bioelectrical impedance analysis; N/A: not available. DICE scores were summarized as follows: †, median (IQR); ‡, mean ± SD; &, mean (95% CI); and #, median ± SD.

## Data Availability

No data were used to support this study.

## Supplementary Materials

Supplementary Materials Supplement Table S1 Normal reference and cut-off for sarcopenia [58, 66, 68–72].
