# Supplementary material for: The Value of Artificial Intelligence-Assisted Imaging in Identifying Diagnostic Markers of Sarcopenia in Patients with Cancer
Source: Dis Markers. 2022 Mar 29;2022:1819841. doi: 10.1155/2022/1819841 (PMC8983171; doi:10.1155/2022/1819841)
Supplement: Supplementary Materials — Supplement Table S1 Normal reference and cut-off for sarcopenia [58, 66, 68–72]. [file 1819841.f1.docx]

Supplement Table S1. Normal reference and cut-off for sarcopenia

|  |  |  |  |  |  | Cut-off (I) | |  | Cut-off (II) | |
| --- | --- | --- | --- | --- | --- | --- | --- | --- | --- | --- |
| Study name Author (year) | Population | Measurements | Men | Women |  | Men | Women |  | Men | Women |
| Kim et al. (2021) | Korea (Healthy) |  |  |  |  |  |  |  |  |  |
|  | Total study participants | SMA/ht2, cm2/m2 | 56.2 ± 7.0 | 42.8 ± 5.2 |  | N/A | N/A |  | N/A | N/A |
|  | Young adult (20-44 years) reference group | SMA/ht2, cm2/m2 | 57.3 ± 7.2 | 42.0 ± 5.5 |  | N/A | N/A |  | N/A | N/A |
| Yoon et al. (2021) | Korea (Living liver donation) |  |  |  |  |  |  |  |  |  |
|  | Total study participants | SMI | 52.89±6.78 | 39.06±5.65 |  | 39.33 | 27.77 |  | 40.96 | 30.6 |
|  | Young adult (20-40 years) reference group | SMI | 53.05±6.63 | 38.78±5.78 |  | 39.79 | 27.22 |  | 42.71 | 30.27 |
| Feng et al. (2019) | China (Living kidney donation) |  |  |  |  |  |  |  |  |  |
|  | Total study participants | SMI | 53.9 ± 6.0 | 43.4 ± 5.3 |  | 41.9 | 32.8 |  | 44.4 | 34.8 |
|  | Young adult (20-50 years) reference group | SMI | 54.0 ± 6.3 | 43.7 ± 5.3 |  | 41.5 | 33.1 |  | N/A | N/A |
| Ufuk et al. (2019) | Tukey (Kidney donation) |  |  |  |  |  |  |  |  |  |
|  | Total study participants | SMI | 50.51±3.95 | 41.32±3.72 |  | 42.6 | 33.9 |  | 45 | 36.1 |
|  | Young adult (20-40 years) reference group | SMI | 51.66±3.58 | 42.28±5.08 |  | N/A | N/A |  | 45.5 | 36.2 |
| Derstine et al. (2018) | USA (Donor) |  |  |  |  |  |  |  |  |  |
|  | 5^th^ percentile of individuals | SMI | N/A | N/A |  | N/A | N/A |  | 45.4 | 34.4 |
| van der Werf et al. (2018) | Caucasian (Donor) |  |  |  |  |  |  |  |  |  |
|  | 5^th^ percentile of individuals | SMI | N/A | N/A |  | N/A | N/A |  | 41.6 | 32 |
| Derstine et al. (2017) | USA (Healthy) |  |  |  |  |  |  |  |  |  |
|  | Total study participants | CSA index | 59.7 ± 7.5 | 47.0 ± 6.5 |  | N/A | N/A |  | 44.6 | 34 |

Cut-off (I): Mean-2SD individual cut-off for sarcopenia; Cut-off (II): 5th percentiles of individuals for sarcopenia.

N/A, not available.
